# The Berlin-Hannover ICANS severity assessment–a novel bedside test to evaluate CAR T-cell-associated neurotoxicity

**DOI:** 10.3389/fneur.2026.1726779

**Published:** 2026-01-21

**Authors:** Lotta Völker, Leonie Müller-Jensen, Sophia Carl, Sandra Nay, Thiemo Malte Möllenkamp, Christian Schultze-Florey, Lea Grote-Levi, Konstantin F. Jendretzky, Franz Felix Konen, Christian Könecke, Matthias Eder, Victoria Panagiota, Florian H. Heidel, Lars Bullinger, Frederik Damm, Mareike Frick, Olaf Penack, Rebecca Ludwig, Eric Anil Buß, Wolfgang Boehmerle, Matthias Endres, Viktoria Gudi, Petra Huehnchen, Thomas Skripuletz, Nora Möhn

**Affiliations:** 1 Department of Neurology, Hannover Medical School, Hannover, Germany; 2 KlinStrucMed Program, Dean’s Office for Academic Career Development, Hannover Medical School, Hannover, Germany; 3 Charité – Universitätsmedizin Berlin, Corporate Member of Freie Universität Berlin and Humboldt Universität zu Berlin, Klinik für Neurologie mit Experimenteller Neurologie, Berlin, Germany; 4 Berlin Institute of Health at Charité–Universitätsmedizin Berlin, Berlin, Germany; 5 Department of Neurology, University Hospital Munster, Munster, Germany; 6 Department of Haematology, Haemostaseology, Oncology and Stem Cell Transplantation, Hannover Medical School, Hannover, Germany; 7 Department of Haematology, Oncology and Tumour Immunology, Charité–Universitätsmedizin Berlin, Berlin, Germany; 8 Center for Stroke Research Berlin, Charité—Universitätsmedizin Berlin, Berlin, Germany; 9 German Center for Neurodegenerative Diseases (DZNE), Berlin, Germany; 10 German Centre for Cardiovascular Diseases (DZHK), Berlin, Germany; 11 German Center for Mental Health (DZPG), Berlin, Germany; 12 Department of Neurology, University Hospital Bonn, Bonn, Germany

**Keywords:** BHISA, CAR T-cell therapy, ICANS, ICE, screening tools

## Abstract

**Background:**

Chimeric antigen receptor (CAR) T-cell therapy has transformed the treatment of refractory hematological malignancies but is frequently complicated by immune effector cell-associated neurotoxicity syndrome (ICANS). Early clinical recognition remains challenging, as the commonly used Immune Effector Cell-Associated Encephalopathy (ICE) score lacks sensitivity for subtle deficits.

**Methods:**

In this prospective bicentric study, 100 patients treated with CAR T-cells at Hannover Medical School and Charité - Universitätsmedizin Berlin underwent systematic neurological assessments using both ICE and the newly developed Berlin-Hannover ICANS Severity Assessment (BHISA). Examinations were performed at baseline prior to CAR T-cell infusion, on day 6–7 (±1 day) post-infusion, and during ICANS episodes. Data on the clinical course, other toxicities, comorbidities, CAR T-cell products, and ICANS treatment were collected.

**Results:**

Thirty-seven patients (37%) developed ICANS, which was associated with preceding cytokine release syndrome and specific CAR T-cell products. While ICE scores clustered at maximum values both at baseline and follow-up, BHISA showed a broader distribution and higher sensitivity to subtle changes. Correlation analyses confirmed agreement between ICE and BHISA, but BHISA captured early cognitive decline more reliably. Receiver operating characteristic analyses demonstrated comparable diagnostic accuracy (BHISA: AUC = 0.783, ICE: AUC = 0,777), with consistently higher sensitivity of BHISA at matched specificity. (Specificity target = 0.7, BHISA sensitivity = 0.743, ICE sensitivity = 0.571; Specificity target = 0.8, BHISA sensitivity = 0.629, ICE sensitivity = 0.571).

**Conclusion:**

BHISA may provide a more sensitive and more differentiated screening tool for ICANS than ICE by incorporating additional cognitive and motor domains, while remaining easy to use. This may enable earlier and more nuanced detection of CAR T related neurotoxicity, potentially improving patient monitoring across a heterogeneous population.

## 1 Introduction

Therapy with chimeric antigen receptor (CAR) T-cells has marked a significant milestone in the treatment of refractory or relapsed hematological malignancies, including diffuse large B-cell lymphoma (DLBCL), multiple myeloma (MM), and acute lymphoblastic B-cell leukemia (B-ALL) (1–3) By genetically modifying autologous T cells so that they express a chimeric receptor capable of recognizing tumor-specific antigens, malignant cells can be specifically targeted and eliminated (4). Pivotal clinical trials have demonstrated substantially higher response rates compared to conventional therapies, with objective response rates (ORR) of up to 74% and complete remission rates (CR) exceeding 50% (1).

However, these significant therapeutic benefits are accompanied by distinct and potentially life-threatening treatment-related toxicities. Cytokine release syndrome (CRS) and immune effector cell-associated neurotoxicity syndrome (ICANS) are the two most relevant adverse events following CAR T-cell therapy (5). CRS is a systemic inflammatory reaction driven by excessive cytokine release and is typically manageable with the interleukin-6 receptor antagonist tocilizumab and corticosteroids. In contrast, the pathophysiology and optimal management of ICANS are less well understood (6–9). Currently, no specific treatment for ICANS is available, and corticosteroids represent the mainstay of first-line therapy (10).

Clinically, ICANS typically occurs between day +2 and +11 following CAR T-cell infusion and presents with a broad spectrum of neurological symptoms. Early signs may include tremor or psychomotor slowing, while severe cases can involve aphasia, seizures, altered consciousness, or, in rare cases, fatal cerebral edema (7, 11, 12). Identified risk factors for ICANS include a high tumor burden, higher CAR T-cell doses, a CAR construct comprising a CD28 co-stimulatory domain, and presence of CRS (7, 12, 13). In addition, pre-existing neurological disorders may increase the risk of developing ICANS (13).

To date, the Immune Effector Cell-Associated Encephalopathy (ICE) score is the most commonly used tool for the clinical assessment of ICANS and is part of the ASTCT Consensus Criteria for grading of ICANS. It evaluates five domains: orientation, object naming, handwriting, attention and the ability to follow commands (14). While the ICE score enables quick standardized documentation of neurological symptoms, it may have notable limitations in sensitivity and differentiation (4, 15–17). In some cases, patients exhibited early neurotoxic signs without any concurrent drop in the ICE score following CAR T-cell treatment. Complex or subtle cognitive deficits may not be recognized, which carries the risk of under- or misdiagnosis in the early stages of neurotoxicity (16, 18). A limited number of research groups addressed the challenge of improving early detection of ICANS. The CART neurotoxicity scale (CART-NS) was presented as a potential tool, which demonstrated promise in a small cohort of 12 patients. In that study, the Stroop test, Oral Symbol Digit (OSD) test, and Paced Visual Serial Addition Test (PVSAT) were found to be particularly predictive of early neurotoxicity (17).

Based on our clinical experience and a literature review, we developed a novel scoring system, the Berlin-Hannover ICANS Severity Assessment (BHISA), which builds upon and extends the domains assessed by the ICE score. In addition to orientation, object naming, and attention, BHISA incorporates measures of verbal fluency, working memory, extended executive function assessment, and motor skills, aiming to better capture the range of ICANS-related neurocognitive symptoms, while maintaining a simplistic nature and completion in a limited timeframe to ensure feasibility in clinical routine.

In the present study, we neurologically monitored 100 patients who underwent CAR T-cell therapy, applying both the ICE and BHISA scoring systems. Our objective was to evaluate the diagnostic performance of BHISA as a screening instrument for ICANS in comparison with ICE.

## 2 Methods

### 2.1 Study design

The study was approved at Hannover Medical School (MHH) and Charité – Universitätsmedizin Berlin (Charité), respectively (ethics approval: BO_S_2023 for MHH, EA1/247/22 for Charité) and registered with the German Clinical Trials Register (DRKS00031604). All patients consented to study participation prior to any study procedure. Overall, a total of 100 patients who underwent CAR T-cell therapy between 2022 and 2025 at either Hannover Medical School (MHH) or Charité - Universitätsmedizin Berlin were included in this prospective, bicentric non-interventional, longitudinal observational study with repeated neurocognitive assessments over time. Patients treated with commercially available CAR T-cell products (idecabtagene vicleucel, axicabtagene ciloleucel, lisocabtagene maraleucel, tisagenlecleucel, brexucabtagene autoleucel, and ciltacabtagene autoleucel) as well as an in-house CAR T-cell product manufactured using CliniMACS Prodigy System technology, were eligible for inclusion. Underlying diagnoses comprised multiple myeloma, diffuse large B-cell lymphoma, primary CNS lymphoma, mantle cell lymphoma, follicular lymphoma, acute lymphoblastic leukemia, primary mediastinal B-cell lymphoma, high-grade B-cell lymphoma, and systemic sclerosis with pulmonary involvement. One patient included in this cohort has been individually presented in a separate case report, which is currently under revision.

Prior to CAR T-cell infusion, each patient received a standardized lymphodepleting chemotherapy regimen consisting of fludarabine and cyclophosphamide. Patients were monitored for at least 10 days following CAR T-cell infusion.

Demographic data including age, sex, underlying disease, and comorbidities were collected for all participants. Race and ethnicity data were not collected. Information on administered CAR T-cell dose was available for 82 patients.

### 2.2 Diagnostic procedures

Patients underwent comprehensive neurological monitoring before and after CAR T-cell therapy. At both study sites, cognitive deficits were assessed daily during the first week post-infusion using the ICE score. Neurological assessments and cognitive evaluations using the BHISA score was performed at baseline and on day +6 (± 1 day) after infusion at MHH and at baseline and on day +7 (± 1 day) at Charité, shown below as baseline and Follow-up (FU). In addition, ICANS episode-specific assessments were performed starting on the day of the first clinically observed neurological symptom. Furthermore, blood samples were obtained at both time points to enable the investigation of potential biomarkers in further analyses. ICANS grading followed the ASTCT Consensus Criteria (14). Declines in ICE or BHISA scores were classified as ICANS only when accompanied by a compatible clinical syndrome and not attributable to alternative causes such as sedation, metabolic abnormalities, or pre-existing cognitive impairment. Electroencephalography (EEG) recordings were acquired with surface electrodes and photic stimulation according to standardized protocols (19), using the international 10/20 electrode montage (20).

### 2.3 Score development

Given the limitations of the existing ICE score in capturing the full clinical spectrum of ICANS, particularly executive dysfunction, tremor, and apraxia, we developed a novel neurotoxicity scoring system. This score was constructed based on our clinical experience with affected patients. Key domains included orientation, attention, verbal fluency, working memory, and executive functions, providing a more granular and clinically relevant assessment of neurocognitive impairment, particularly in early or subtle cases. Frontal lobe dysfunction was examined using the palmomental reflex, which was classified as positive if present unilaterally or bilaterally and was used as an indicator of frontal lobe dysfunction; however, none of the patients in our cohort exhibited a positive reflex. In addition, writing performance was assessed qualitatively, and any clearly abnormal writing pattern, including tremulous writing or apraxic features, was classified as pathological. Specific writing abnormalities (e.g., paligraphia) were not systematically documented and could therefore not be analyzed (21). The scale’s maximum of 17 points allows for finer discrimination than the 10-point ICE score.

### 2.4 Statistical analysis

All statistical analyses were performed in R (version 4.4.3) using RStudio IDE (22). Raw data import, cleaning, and typecasting were handled with the packages readxl (23), tidyverse (24), janitor (25), and naniar (26). Descriptive statistics were calculated for exploratory data analysis. Continuous variables are presented as mean ± standard deviation, categorical variables as absolute and relative frequencies (%). Between-group comparisons (ICANS vs. non-ICANS) were employed nonparametric Wilcoxon tests for continuous variables and chi-square tests for categorical variables, with a two-sided *p* < 0.05 indicating statistical significance. The results were compiled in a structured overview table. This was converted into a publication-ready table format using the flextable (24) and officer (27) packages. Spearman’s rank correlation coefficient was used to evaluate the association between BHISA and ICE scores. Scatter plots with linear regression lines and histograms illustrating ICE and BHISA at before and after CAR T-cell therapy, as well as the differences in ICE and BHISA scores between the two time points were created using ggpubr (28) and ggplot2 (29). The cowplot (30) package was used to combine several figures in a common layout. Predictive performance of the BHISA score for ICANS onset was assessed by receiver operating characteristic (ROC) analysis with 2,000 bootstrap replicates using the pROC package (31). Optimal cut-off values were identified by the Youden index and the “closest top-left” criterion. Corresponding sensitivity and specificity values were calculated for each threshold, and the ROC curve was visualized with ggplot2. This was followed by a direct head-to-head comparison of BHISA and ICE at FU. ROC curves were generated for both instruments using a complete-case dataset. The AUCs with 95% confidence intervals were calculated using the DeLong method. A paired comparison of the two AUCs was then performed using the DeLong test for correlated ROC curves to statistically assess whether the overall diagnostic accuracy differed between BHISA and ICE. Sensitivity, specificity and the Youden index were calculated for both instruments at predefined score thresholds. In addition, the sensitivity of both scores was compared at predefined specificity levels (0.50–1.00 in 0.05 increments). For each specificity target, the closest observed threshold was used, and the corresponding sensitivity was calculated. To directly compare sensitivity between instruments while accounting for paired observations, analyses were restricted to the subgroup of patients with ICANS, a paired comparison of sensitivities was performed using the McNemar test. The results of the cut-off determinations and sensitivity comparisons were presented in tabular form and in composite figures with curve plots and parameter overviews.

## 3 Results

### 3.1 Patient characteristics

A total of 100 patients were included in the study. Data on demographics and clinical characteristics is outlined in Table 1. In brief, 37 (37%) patients developed ICANS and 63 (63%) did not. All treated patients with ICANS received dexamethasone, although cumulative dosing data were not consistently available; two patients with ICANS grade 1 did not receive corticosteroids. Additional treatments included prednisolone, levetiracetam, or anakinra in selected cases. CRS occurred in 77 patients (77%) and was significantly more frequent in those who developed ICANS (*p* < 0.001). ICANS occurred at a median of days 6–7 after infusion; however, one individual case manifested significantly later, at day +25, and was classified as ICANS grade 5 later on. Most patients were treated for either DLBCL (35/100, 35%) or MM (36/100, 36%). The most commonly used CAR T-cell products were axicabtagene ciloleucel and ciltacabtagene autoleucel, together accounting for 60% of all infusions. Patients who developed ICANS were significantly more likely to have been treated with axicabtagene ciloleucel (*p* = 0.002) and brexucabtagene autoleucel (*p* = 0.04) compared to patients without ICANS. In contrast, patients who developed ICANS received significantly less frequently ciltacabtagene autoleucel to (*p* = 0.001). Regarding underlying tumor diseases, ICANS was less common in patients with MM (*p* < 0.001), but more frequent in those with DLBCL (*p* = 0.016) or mantle cell lymphoma (*p* = 0.026). No significant differences were observed between ICANS and non-ICANS groups with respect to age or average CAR T-cell dose per kilogram body weight (*p* = 0.462).

**Table 1:** Patient characteristics at baseline.

Characteristics	Total cohort (*n* = 100)	No ICANS (*n* = 63)	ICANS (*n* = 37)	*p*-value	OR	95% CI
Sex						
Male	64 (64%)	35 (55.6%)	29 (78.4%)	**0.031**	2.87	[1.07; 8.45]
Female	36 (36%)	28 (44.4%)	8 (21.6%)	**0.031**	0.35	[0.12; 0.94]
Age	61.07 ± 12.03	62.14 ± 10.52	59.24 ± 14.20	0.504		
Average T-cell dosage^#^	52,666,432.93 ± 81,671,460.30	47,303,490.57 ± 81,076,310.00	62,467,672.41 ± 83,273,018.65	0.462		
Tumor disease						
MM	36 (36%)	32 (50.8%)	4 (10.8%)	**< 0.001**	0.12	[0.03; 0.39]
DLBCL	35 (35%)	16 (25.4%)	19 (51.4%)	**0.010**	3.06	[1.21; 7.99]
PCNSL	1 (1%)	0 (0%)	1 (2.7%)	0.370		
Mantle cell lymphoma	6 (6%)	1 (1.6%)	5 (13.5%)	**0.025**	9.47	[1.00; 464.19]
Follicular lymphoma	8 (8%)	6 (9.5%)	2 (5.4%)	0.707		
ALL	6 (6%)	2 (3.2%)	4 (10.8%)	0.190		
PMBCL	2 (2%)	2 (3.2%)	0 (0%)	0.529		
DLBCL + transformed FL	2 (2%)	2 (3.2%)	0 (0%)	0.529		
High-grade BCL	3 (3%)	1 (1.6%)	2 (5.4%)	0.553		
Other^*^	1 (1%)	1 (1.6%)	0 (0%)	1.000		
CAR T-cell product						
Idecabtagen Vicleucel	5 (5%)	5 (7.9%)	0 (0%)	0.154		
Axicabtagen Ciloleucel	26 (26%)	9 (14.3%)	17 (45.9%)	**0.001**	4.91	[1.74; 14.77]
Lisocabtagene maraleucel	21 (21%)	13 (20.6%)	8 (21.6%)	1.000		
Tisagenlecleucel	5 (5%)	4 (6.3%)	1 (2.7%)	0.648		
Brexucabtagen autoleucel	10 (10%)	3 (4.8%)	7 (18.9%)	**0.037**	4.51	[0.95; 28.99]
Ciltacabtagene autoleucel	30 (30%)	26 (41.3%)	4 (10.8%)	**0.001**	0.17	[0.04; 0.57]
Other^**^	1 (1%)	1 (1.6%)	0 (0%)	1.000		
Outcome						
PR	15 (15%)	10 (15.9%)	5 (13.5%)	0.781		
SD	10 (10%)	5 (7.9%)	5 (13.5%)	0.494		
Death	16 (16%)	7 (11.1%)	9 (24.3%)	0.099		
PD	6 (6%)	2 (3.2%)	4 (10.8%)	0.193		
CR	15 (15%)	10 (15.9%)	5 (13.5%)	0.781		
VGPR	6 (6%)	5 (7.9%)	1 (2.7%)	0.406		
Metabolic progressive disease	1 (1%)	1 (1.6%)	0 (0%)	1.000		
NA	27 (27%)	20 (31.7%)	7 (18.9%)	0.240		
Neurological comorbidities						
No	68 (68%)	43 (68.3%)	25 (67.6%)	1.000		
Yes	32 (32%)	20 (31.7%)	12 (32.4%)	1.000		
Neurological examination at baseline^##^						
Neuropathy^***^						
Yes	56 (56%)	30 (47.6%)	26 (70.3%)	**0.045**	3.25	[0.99; 12.72]
No	24 (24%)	19 (30.2%)	5 (13.5%)	**0.045**	0.31	[0.08; 1.01]
Other neurological findings^***^						
Yes	32 (32%)	22 (34.9%)	10 (27.0%)	0.350		
No	48 (48%)	27 (42.9%)	21 (56.8%)	0.350		
CRS^****^						
Yes	77 (77%)	41 (65.1%)	36 (97.3%)	**< 0.001**	18.92	[2.78; 815.66]
No	22 (21%)	22 (34.9%)	1 (2.7%)	**< 0.001**	0.05	[0.00; 0.36]
CRS grade	1.62 ± 0.74	1.41 ± 0.55	1.86 ± 0.87	**0.010**	2.70	[−1.00; −0.00]
ICANS grade	2.11 ± 1.24	-	2.11 ± 1.24			
ICE						
Baseline	9.87 ± 0.53	9.95 ± 0.28	9.73 ± 0.77	**0.022**	NA^###^	[−0.00; 0.00]
FU	9.31 ± 1.93	9.95 ± 0.28	8.19 ± 2.86	**< 0.001**	0.09	[0.00; 1.00]
ICANS (lowest score)	6.00 ± 3.44	-	6.00 ± 3.44			
ICANS Mean	7.04 ± 2.36	-	7.04 ± 2.36			
BHISA						
Baseline	15.64 ± 1.51	15.84 ± 1.24	15.30 ± 1.84	0.217		
FU	14.55 ± 3.35	15.80 ± 1.22	12.37 ± 4.58	**< 0.001**	0.53	[1.00; 3.00]
ICANS (lowest score)	10.53 ± 4.49	–	10.53 ± 4.49			
ICANS Mean	11.57 ± 3.47	–	11.57 ± 3.47			

Data are presented as mean ± SD or *n* (%). MM, Multiple myeloma; DLBCL, Diffuse Large B-cell lymphoma; PCNSL, Primary central nervous system lymphoma; ALL, Acute lymphoblastic leukemia; PMBCL, Primary mediastinal B-cell lymphoma; BCL, B-cell lymphoma; FL, Follicular lymphoma; PR, Partial response; SD, Stable disease; PD, Progressive disease; CR, Complete response; VGPR, Very good partial response; CRS, Cytokine release syndrome; ICANS, Immune effector cell-associated neurotoxicity syndrome; ICE, Immune effector cell-associated encephalopathy score; BHISA, Berlin-Hannover ICANS severity assessment; FU, Follow-up; OR, Odds ratio; CI, Confidence interval.*Systemic sclerosis with pulmonary involvement; **In-house cell preparation based on CliniMACS Prodigy System technology; ***Data on neurological examination prior to the start of therapy was available for 80 patients; ****In one patient no data on CRS development was applicable; ^#^ Data on average T-cell dosage was available for 82 patients; ^##^Details are shown in Supplementary Table 1; ^###^ Odds ratios for ICE baseline were not estimable owing to minimal variance and quasi-complete separation. Values in bold indicate a significant correlation.

### 3.2 Neurological findings at baseline

Many patients already exhibited neurological symptoms prior to CAR T-cell therapy or had a pre-existing neurological diagnosis. Fifty-six patients (62.2%) presented with peripheral neuropathy, 31 patients (34.4%) with other neurological abnormalities, and 32 patients (32.3%) had a known neurological diagnosis. Peripheral neuropathy at baseline was significantly more frequent among patients who later developed ICANS (*p* = 0.045) (Table 1). In contrast, the prevalence of other neurological symptoms such as ataxic gait, tremor, or cranial nerve palsies was comparable between both groups (Supplementary Table 1).

### 3.3 Comparison of BHISA and ICE scores

**Figure 1:**
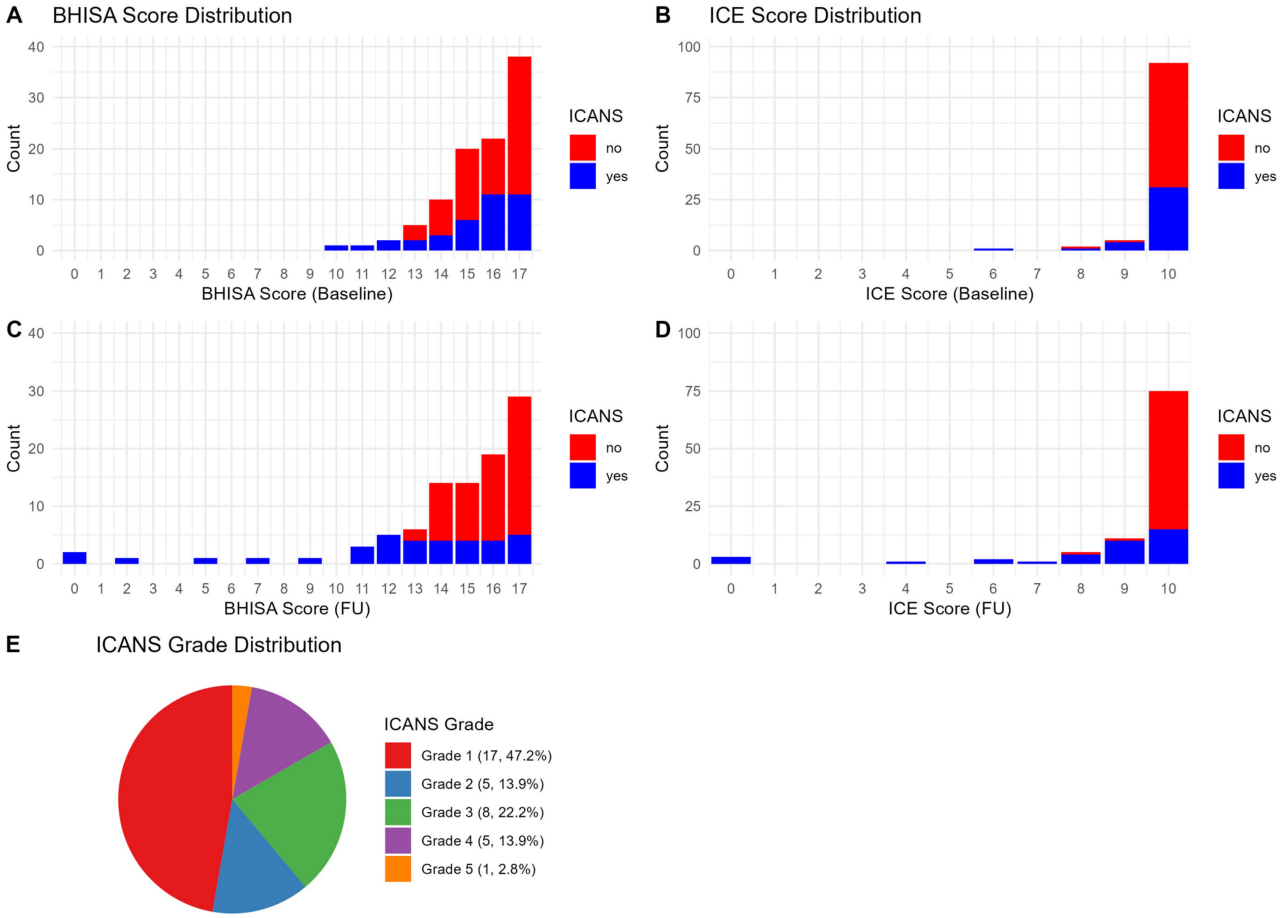
**(A–E)** Distribution of BHISA scores **(A,C)** and ICE scores **(B,D)** at baseline (prior to CAR-T) and at FU after CAR T-cell infusion in patients with and without ICANS. Distribution of ICANS severity grades in the study population at the time of maximum severity **(E)**. ICE, Immune Effector Cell-Associated Encephalopathy Score; BHISA, Berlin-Hannover ICANS Severity Assessment; ICANS, Immune Effector Cell-Associated Neurotoxicity Syndrome; FU, Follow-up.

At baseline, ICE and BHISA scores were collected from 100 and 99 patients, respectively. At FU, ICE score was performed in 98 patients and BHISA score in 96 patients. Figure 1 illustrates the distribution of ICE and BHISA scores at the respective time points. At baseline, the vast majority of patients achieved the maximum ICE score of 10, a pattern that remained largely unchanged at follow-up. In contrast, although many patients reached the maximum BHISA score of 17 at baseline, the distribution of BHISA scores was notably broader at both time points. One patient presented with an ICE score of 8 already at baseline; as the score did not decline after CAR T-cell infusion, this was not classified as ICANS according to ASTCT criteria.

For the subset of patients assessed during active ICANS (*n* = 15), BHISA and ICE score distributions at symptom onset are shown descriptively in Supplementary Figures 2, 3. Due to the small sample size, no inferential analyses were performed.

**Figure 2:**
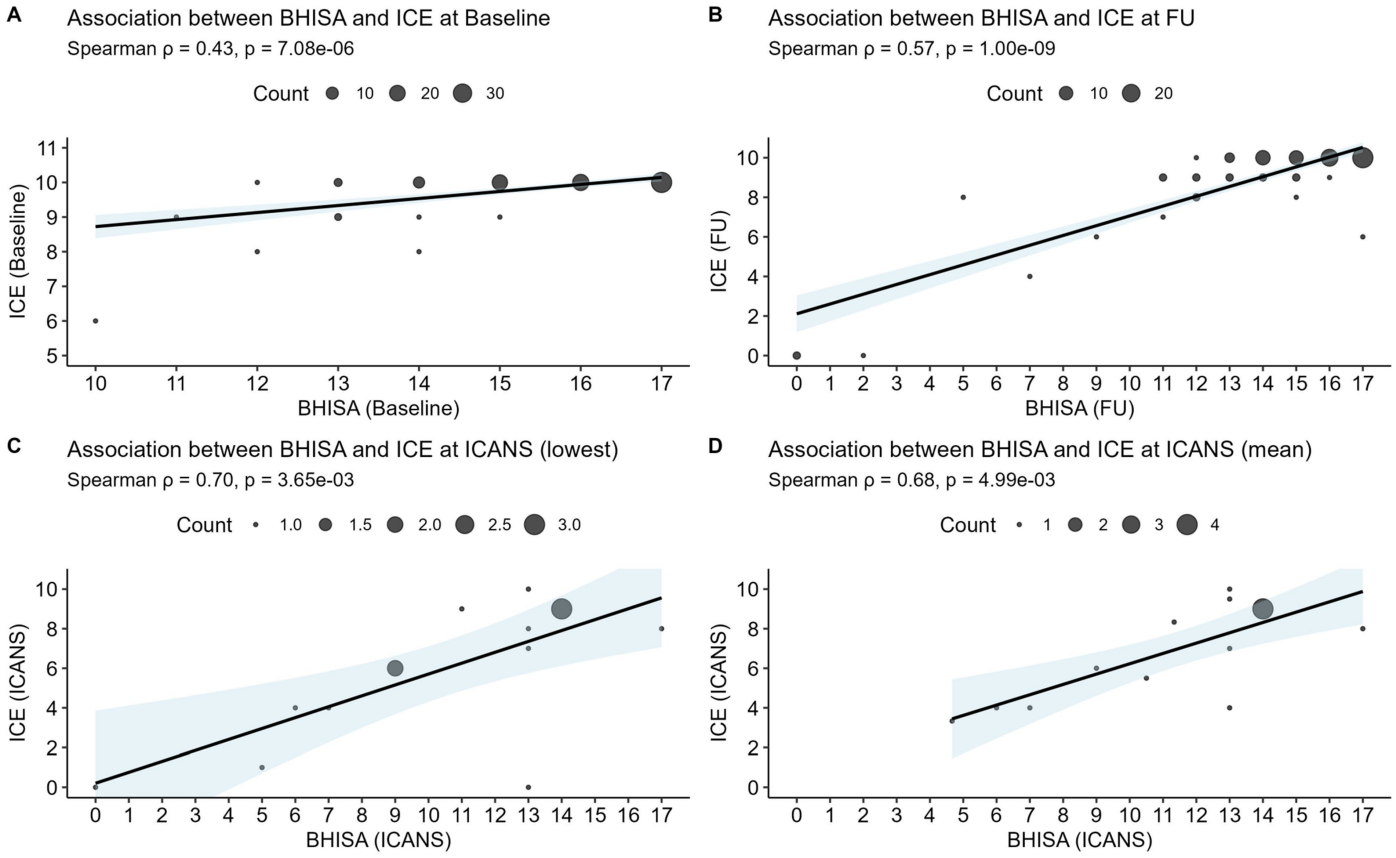
Spearman’s correlation analysis of the ICE and BHISA scores at baseline **(A)**, at FU **(B)**, and during ICANS, showing both the lowest recorded scores **(C)** and the mean values throughout ICANS **(D)**. The 95% confidence interval is illustrated in blue. ICE, Immune Effector Cell-Associated Encephalopathy Score; BHISA, Berlin-Hannover ICANS Severity Assessment; ICANS, Immune Effector Cell-Associated Neurotoxicity Syndrome.

To explore the relationship between the two assessment tools, we conducted a correlation analysis using Spearman’s rank correlation coefficient, as the data were not normally distributed. Figure 2 demonstrates a positive correlation between ICE and BHISA scores across different time points. The correlation is moderate at baseline (*p* = 0.43) and becomes stronger by FU (*p* = 0.57). An even more pronounced correlation emerges during episodes of ICANS. As ICANS-related scores were sometimes recorded on different days, Figure 2C displays the lowest/worst recorded ICE and BHISA scores per patient (*p* = 0.70), whereas Figure 2D presents the mean of all score values documented during the ICANS period (*p* = 0.68). It should be noted, however, that only 15 patients had both ICE and BHISA scores available during an ICANS episode.

**Figure 3:**
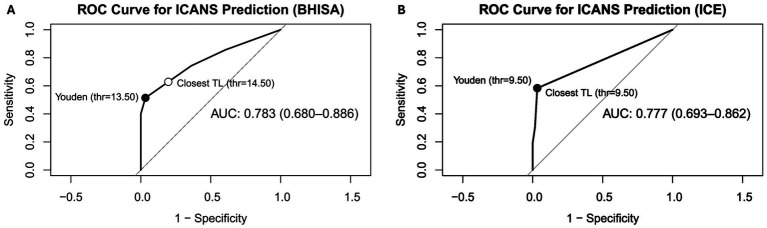
**(A,B)** ROC curves for discriminating between patients with ICANS (*n* = 37) based on the ICE score **(A)** and the BHISA score **(B)** at FU after CAR T-cell infusion. Youden index suggested an optimal cut-off of 13.5 (sensitivity = 51.4% and specificity = 96.7%) for BHISA score and the closest-top-left method suggested a cut-off of 14.5 (sensitivity = 62.9% and specificity = 80%). For the ICE score the Youden index and the closest-top-left method identified an optimal cut-off of 9.5 (sensitivity = 58.3% and specificity = 96.8%). AUC, Area under the curve; ICE, Immune Effector Cell-Associated Encephalopathy Score; BHISA, Berlin-Hannover ICANS Severity Assessment.

ROC analysis for the ICE score at FU demonstrated an area under the curve (AUC) of 0.777 (95% confidence interval: 0.693–0.862). Both the Youden index and the closest-top-left method identified an optimal cut-off of 9.5, corresponding to a sensitivity of 58.3% and a specificity of 96.8% (Figure 3).

For the BHISA score at FU, ROC analysis yielded a comparable AUC of 0.783 (95% confidence interval: 0.68–0.886). The Youden index suggested an optimal cut-off of 13.5, achieving a sensitivity of 51.4% and a specificity of 96.7%. In contrast, the closest-top-left method proposed a slightly higher cut-off of 14.5, resulting in improved sensitivity (62.9%) at the expense of reduced specificity (80%).

### 3.4 ICANS follow-up – operating characteristics and paired sensitivity tests

**Figure 4:**
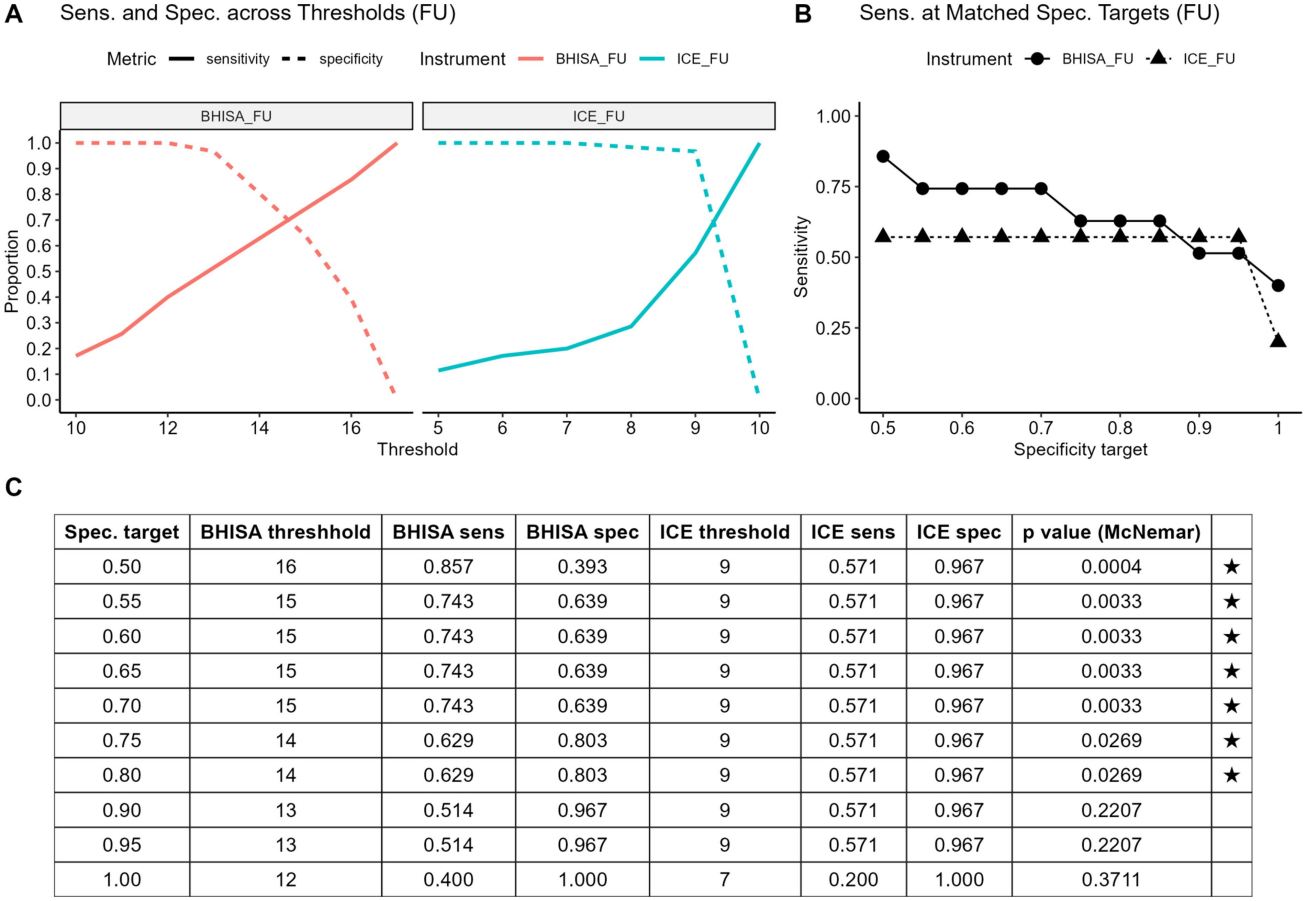
**(A–C)** Sensitivity and specificity of BHISA and ICE for different cut-offs **(A)**. Sensitivity of BHISA and ICE at identical, stepwise increasing specificity targets ranging from 0.5 to 1.0 **(B)**. All data on sensitivity and specificity at different cut-offs are presented in tabular form **(C)**. ICE: Immune Effector Cell-Associated Encephalopathy Score; BHISA: Berlin-Hannover ICANS Severity Assessment.

The BHISA score demonstrated diagnostic performance comparable to the established ICE score in terms of overall accuracy. Given that the BHISA score was developed primarily as a screening tool for ICANS, the analysis focused on its sensitivity across various specificity thresholds (Figure 4A). To assess this, we compared the sensitivity of both scores at predefined specificity levels ranging from 50 to 100%. For each level, the corresponding closest threshold was identified, and sensitivity was calculated accordingly. Due to the integer scoring scale, several specificity targets result in the same cut-off values. Sensitivities were then compared using the McNemar test. When sensitivity was compared at identical specificity targets, the BHISA score demonstrated significantly higher sensitivity for ICANS detection than the ICE score across most specificity levels, particularly between 50 and 80% (*p* < 0.05; Figure 4B). This indicates improved identification of ICANS without loss of specificity. The associated thresholds, sensitivities, and specificities are summarized in Figure 4C.

**Figure 5:**
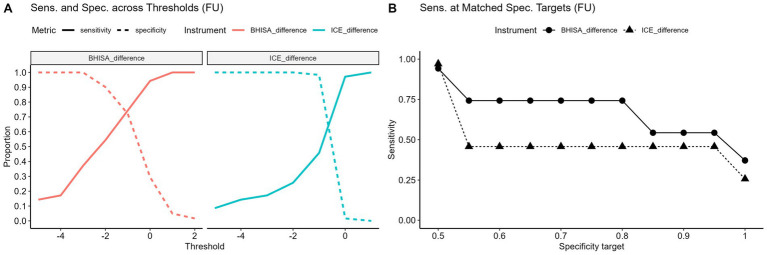
**(A,B)** Sensitivity and specificity at varying point differences between baseline and FU (*Δ* = FU-baseline) for different cut-offs **(A)**. Negative values indicate a decrease from baseline. Sensitivity of baseline-to-follow-up point differences in BHISA and ICE at identical, stepwise increasing specificity targets ranging from 0.5 to 1.0 **(B)**. ICE, Immune effector cell-associated encephalopathy score; BHISA, Berlin-Hannover ICANS severity assessment.

This effect is particularly evident when baseline values are taken into account and delta values are evaluated from baseline to FU. In Figure 5A, point differences between the two time points were used as cut-off values to determine sensitivity and specificity. As shown in Figure 5B, this approach further supports the superior sensitivity of the BHISA score. When comparing point differences in sensitivity at matched specificity targets, BHISA outperformed ICE across the full range of values examined, maintaining higher sensitivity even at high specificity levels. A detailed overview of score dynamics in patients with and without ICANS is provided in the Supplementary Figure 1.

## 4 Discussion

CAR T-cell therapy has revolutionized the treatment of hematological malignancies. However, its clinical success is tempered by side effects, most notably CRS and ICANS. Given the potentially severe consequences of ICANS and the lack of specific treatments, timely and accurate recognition and prediction remains a central clinical challenge. In this study, we aimed to systematically characterize neurological complications following CAR T-cell therapy and to evaluate a newly developed screening tool, the BHISA, in comparison to the established ICE score. Our study yielded the following findings: Both scores demonstrated overall equivalence. Compared with the ICE score, the BHISA provides greater scale granularity and a broader coverage of the symptom spectrum, enhancing its discriminatory capacity and allowing for a more nuanced characterization of neurological deficits. This improved resolution facilitates the detection of subtle abnormalities, giving the BHISA higher sensitivity at equal specificity and making it more suitable as a screening tool. In clinical practice, such performance advantages are particularly valuable when combined with rapid and flexible applicability. With an administration time of approximately 3–5 min, BHISA represents a time-efficient assessment tool that can be conducted not only by physicians but also by trained nursing staff or paramedics.

In our cohort of 100 patients treated with CAR T-cell therapy, 37 developed ICANS of grade 1–5. The average onset of ICANS symptoms was observed around day 6–7 post-infusion, although one case presented much later, on day +109. The overall incidence of ICANS in our cohort appears somewhat higher than reported in pivotal clinical trials (32). This likely reflects the predominant use of axicabtagene ciloleucel and brexucabtagene autoleucel, which carry higher neurotoxicity risks, compared to less frequently used products with lower ICANS rates (33–38). In addition, prior CRS was associated with an increased risk of subsequent ICANS in our cohort, consistent with published data (12, 39). We also observed that patients with pre-existing peripheral neuropathy at baseline were more likely to develop ICANS; however, this association should be interpreted with caution, as neuropathy is rarely analyzed as an independent risk factor in the literature (12). Interestingly, in our cohort male sex was significantly associated with ICANS occurrence. While this observation suggests that sex might influence the ICANS risk, current evidence does not support a consistent sex-specific predisposition (40). It is therefore plausible that the effect observed reflects interactions with other risk factors like the underlying tumor disease or the CAR T-cell product. This differs from COVID-19-associated cytokine syndrome in which male patients are disproportionately affected by cytokine storms (41).

While most patients in our study developed symptoms within the first 6–10 days, isolated late-onset cases suggest that a more extended surveillance period may be warranted, especially in high-risk patients. Given this variability, sensitive and broadly applicable screening tools are essential to ensure early detection of neurotoxicity and prevent progression to more severe stages. In this context, assessment tools must strike a balance between specificity and the ability to detect subtle or atypical signs, especially in patients with pre-existing neurological conditions or cognitive deficits. To meet these requirements, we developed the BHISA score as an expanded neurological screening tool. In contrast to the ICE score, the BHISA scale allows for finer differentiation and more comprehensive recording of neurological symptoms. In addition to core elements such as orientation and attention, BHISA also includes verbal fluency, working memory, executive functions and motor skills, enabling a more differentiated assessment of cognitive and behavioral changes. The BHISA score has a significantly broader distribution even before therapy. This could be explained by the presence of pre-existing neurological impairments within the cohort.

BHISA allowed for a more nuanced characterization of neurological deficits in our cohort, particularly in cases with mild or non-specific symptoms. Both scores generally agreed in identifying overlapping aspects of neurological dysfunction and tended to decrease in parallel over the course of neurotoxicity. However, the broader distribution of BHISA scores likely reflects its increased sensitivity to subtle or pre-existing deficits. Despite this broader spectrum, both scores showed global equivalence in terms of diagnostic value, as the AUC results in the ROC analysis did not differ significantly. Importantly, BHISA appears to have greater sensitivity for detecting ICANS with comparable specificity. In our cohort, a BHISA cut-off of 14 points detected ICANS in 21 patients, compared to 17 identified by the ICE score. This might suggest that BHISA could be a suitable bedside screening tool, with a detection performance comparable to ICE and without an increase in false-positive results. In contrast, ICE may be more stringent. It offers high specificity, but at the expense of slightly reduced sensitivity. These results underscore the potential clinical benefit of BHISA as a more sensitive and flexible tool for early detection and monitoring of ICANS, especially in heterogeneous patient groups.

Since some patients already exhibited neurological symptoms prior to CAR T-cell therapy and therefore did not achieve the maximum BHISA score at baseline, we also analyzed score differences between baseline and FU. In this comparison, the BHISA score again proved more sensitive with comparable specificity, showing a significant decline at FU in patients who developed ICANS. Cases without score changes despite ICANS can likely be explained by the timing of symptom onset, which in some patients occurred either before or after FU and was thus not captured within this window. When considering score reductions rather than absolute values, BHISA demonstrated even higher sensitivity than ICE while maintaining comparable specificity. These findings indicate that patient-specific baseline values are relevant for ICANS detection. Whether this concept can be translated into a practical screening approach for routine care remains to be determined and requires prospective, multicenter validation studies. Here, it is important to discuss that the implementation of CAR T-cell therapy in neurology calls for the development of more precise screening tools. For example, the ICE score may not be suitable for diagnosing ICANS in patients with autoimmune encephalitis receiving CAR T-cell treatment, highlighting the need for tailored assessment methods in this population.

In parallel with our efforts, another study group has developed a complementary assessment tool, CART-NS, which focuses on neurocognitive parameters and has shown promising results in a small patient cohort (17). Tests such as Stroop, OSD, and PVSAT can help to further characterize specific cognitive domains affected in early ICANS and could serve as a useful complement to structured clinical scores such as BHISA. However, these tests tend to be more time-intensive and dependent on stringent test procedures, which may limit their practicality, whereas BHISA offers a more streamlined option that is easier to integrate into everyday clinical workflows. Moreover, BHISA can be performed by oncologists as the primary caregivers, which may be a significant practical benefit.

In addition to improved clinical phenotyping, future research should aim to integrate clinical assessment systems with fluid or imaging biomarkers such as cytokine profiles, neurofilament light chain (NfL) levels, or advanced MRI-based techniques (12, 42, 43). Such multimodal approaches could help to better stratify patients according to their risk, monitor disease progression, and potentially guide individualized therapeutic interventions. Ultimately, combining sensitive, domain-specific cognitive assessment tools such as BHISA or CART-NS with objective biomarkers could pave the way for more precise and personalized neurotoxicity management in CAR T-cell therapy.

This study has several limitations. First, neurocognitive testing was conducted at predefined time points that did not always align with the onset or peak of ICANS symptoms, which may have resulted in underestimation or delayed detection of neurotoxicity. Because BHISA was administered on day +6/7, some patients had already experienced and partially recovered from ICANS at the time of assessment. In addition, the observation period was relatively short and did not permit evaluation of long-term neurocognitive outcomes Since the diagnosis of ICANS was based on the ASTCT consensus criteria, which include the ICE score, a direct comparison of diagnostic superiority between BHISA and ICE is limited. An independent gold standard in the form of a validated test or biomarker for the objective confirmation of ICANS is not yet available. Furthermore, although several BHISA items probe executive functioning and frontal network integrity-domains frequently affected in ICANS, item-level impairments were not systematically coded, and domain-specific analyses were therefore not feasible. Future studies should include structured, domain-level scoring to delineate which BHISA components contribute most strongly to detecting early neurotoxicity. A statistical limitation is that only integer cut-offs can be applied, preventing the use of the optimal threshold values identified in the ROC analysis. Finally, sensitivity comparisons were based on specificity levels that were only approximate. As both scores allow only whole-number values, cut-offs and sensitivities had to be calculated for predefined specificity targets, which could only be approximated rather than matched exactly. It also emerged that identical cut-off values resulted for several specificity targets.

## 5 Conclusion

In this prospective bicentric cohort, the novel BHISA score provided a more differentiated and sensitive bedside tool for detecting neurocognitive changes following CAR T-cell therapy than the widely used ICE score. BHISA captured subtle deficits, demonstrated higher sensitivity at comparable specificity, and allowed a broader characterization of neurological symptoms, particularly in patients with pre-existing neurological abnormalities. While both scores showed comparable overall diagnostic accuracy, BHISA may represent a more suitable bedside screening instrument and could facilitate earlier recognition and monitoring of ICANS in clinical practice. Integration of BHISA into routine assessment could help to identify at-risk patients earlier, guide timely therapeutic interventions, and support more personalized management of CAR T-cell associated neurotoxicity. Prospective multicenter validation studies are warranted to confirm these findings and to further refine screening strategies.

## Data Availability

The raw data supporting the conclusions of this article will be made available by the authors, without undue reservation.

## References

[1] Locke FL Neelapu SS Bartlett NL Siddiqi T Chavez JC Hosing CM . Phase 1 results of ZUMA-1: a multicenter study of KTE-C19 anti-CD19 CAR t cell therapy in refractory aggressive lymphoma. Mol Ther. (2017) 25:285–95. doi: 10.1016/j.ymthe.2016.10.020, 28129122 PMC5363293

[2] Schuster SJ Svoboda J Chong EA Nasta SD Mato AR Anak Ö . Chimeric antigen receptor T cells in refractory B-cell lymphomas. N Engl J Med. (2017) 377:2545–54. doi: 10.1056/NEJMoa1708566, 29226764 PMC5788566

[3] Brudno JN Kochenderfer JN. Chimeric antigen receptor T-cell therapies for lymphoma. Nat Rev Clin Oncol. (2018) 15:31–46. doi: 10.1038/nrclinonc.2017.128, 28857075 PMC12145160

[4] Kazzi C Kuznetsova V Siriratnam P Griffith S Wong S Tam CS . Cognition following chimeric antigen receptor T-cell therapy: a systematic review. J Autoimmun. (2023) 140:103126. doi: 10.1016/j.jaut.2023.103126, 37837807

[5] Morris EC Neelapu SS Giavridis T Sadelain M. Cytokine release syndrome and associated neurotoxicity in cancer immunotherapy. Nat Rev Immunol. (2022) 22:85–96. doi: 10.1038/s41577-021-00547-6, 34002066 PMC8127450

[6] Titov A Petukhov A Staliarova A Motorin D Bulatov E Shuvalov O . The biological basis and clinical symptoms of CAR-T therapy-associated toxicites. Cell Death Dis. (2018) 9:897. doi: 10.1038/s41419-018-0918-x, 30181581 PMC6123453

[7] Santomasso BD Park JH Salloum D Riviere I Flynn J Mead E . Clinical and biologic correlates of neurotoxicity associated with CAR T cell therapy in patients with B-cell acute lymphoblastic leukemia (B-ALL). Cancer Discov. (2018) 8:958–71. doi: 10.1158/2159-8290.CD-17-1319, 29880584 PMC6385599

[8] Santomasso BD Nastoupil LJ Adkins S Lacchetti C Schneider BJ Anadkat M . Management of immune-related adverse events in patients treated with chimeric antigen receptor T-cell therapy: ASCO guideline. J Clin Oncol. (2021) 39:3978–92. doi: 10.1200/JCO.21.01992, 34724386

[9] Diorio C Shraim R Myers R Behrens EM Canna S Bassiri H . Comprehensive serum proteome profiling of cytokine release syndrome and immune effector cell-associated neurotoxicity syndrome patients with B-cell ALL receiving CAR T19. Clin Cancer Res. (2022) 28:3804–13. doi: 10.1158/1078-0432.CCR-22-0822, 35705524 PMC9444956

[10] Neelapu SS Tummala S Kebriaei P Wierda W Gutierrez C Locke FL . Chimeric antigen receptor T-cell therapy - assessment and management of toxicities. Nat Rev Clin Oncol. (2018) 15:47–62. doi: 10.1038/nrclinonc.2017.148, 28925994 PMC6733403

[11] Maude SL Laetsch TW Buechner J Rives S Boyer M Bittencourt H . Tisagenlecleucel in children and young adults with B-cell lymphoblastic leukemia. N Engl J Med. (2018) 378:439–48. doi: 10.1056/NEJMoa1709866, 29385370 PMC5996391

[12] Gust J Hay KA Hanafi L Li D Myerson D Gonzalez-Cuyar LF . Endothelial activation and blood-brain barrier disruption in neurotoxicity after adoptive immunotherapy with CD19 CAR-T cells. Cancer Discov. (2017) 7:1404–19. doi: 10.1158/2159-8290.CD-17-0698 29025771 PMC5718945

[13] Butt OH Zhou AY Ances BM DiPersio JF Ghobadi A. A systematic framework for predictive biomarkers in immune effector cell-associated neurotoxicity syndrome. Front Neurol. (2023) 14:14. doi: 10.3389/fneur.2023.1110647 PMC9969296 36860569

[14] Lee DW Santomasso BD Locke FL Ghobadi A Turtle CJ Brudno JN . ASTCT consensus grading for cytokine release syndrome and neurologic toxicity associated with immune effector cells. Biol Blood Marrow Transplant. (2019) 25:625–38. doi: 10.1016/j.bbmt.2018.12.758 30592986 PMC12180426

[15] Vonberg FW Malik I O'Reilly M Hyare H Carr AS Roddie C. Neurotoxic complications of chimeric antigen receptor (CAR) T-cell therapy. J Neurol Neurosurg Psychiatry. (2025) 96:665–78. doi: 10.1136/jnnp-2024-333924 40185628

[16] Sales C Anderson MA Kuznetsova V Rosenfeld H Malpas CB Roos I . Patterns of neurotoxicity among patients receiving chimeric antigen receptor T-cell therapy: a single-Centre cohort study. Euro J of. Neurology. (2024) 31:174. doi: 10.1111/ene.16174 PMC11235605 38085272

[17] Suresh A Wishart HA Arslan MN Lizcano RA Shah PS PonnamReddy S . Novel neurocognitive testing tool for early neurotoxicity detection following anti-CD19 and anti-BCMA chimeric antigen receptor (CAR) T-cell therapy: a pilot study. Clin Lymphoma Myeloma Leuk. (2025) 25:365–78. doi: 10.1016/j.clml.2024.12.011 39814673 PMC13086173

[18] Herr MM Chen GL Ross M Jacobson H McKenzie R Markel L . Identification of neurotoxicity after chimeric antigen receptor (CAR) T cell infusion without deterioration in the immune effector cell encephalopathy (ICE) score. Biol Blood Marrow Transplant. (2020) 26:e271–4. doi: 10.1016/j.bbmt.2020.07.031 32736009

[19] Suhs K. W. Wegner F. Skripuletz T. Trebst C. Tayeb S. B. Raab P., &, Stangel M. Heterogeneity of clinical features and corresponding antibodies in seven patients with anti-NMDA receptor encephalitis. Exp Ther Med 2015;10:1283–1292. doi: 10.3892/etm.2015.2689, . 26622479 PMC4577954

[20] Klem GH Lüders HO Jasper HH Elger C. The ten-twenty electrode system of the international federation. The International Federation of Clinical Neurophysiology. Electroencephalogr Clin Neurophysiol Suppl. (1999) 52:3–6. 10590970

[21] Fontanelli L Pizzanelli C Pizzanelli C Milano C Cassano Cassano R Galimberti S . Pre-existing frontal lobe dysfunction signs as predictors of subsequent neurotoxicity in CAR T cell therapy: insights from a case series. Neurol Sci. (2023) 44:3291–7. doi: 10.1007/s10072-023-06841-6, 37160803 PMC10170036

[22] Posit Team. *RStudio: Integrated Development Environment for R*. (2025). Available online at: http://www.posit.co/.

[23] Wickham H. *Readxl: Read Excel Files*. R package version 1.4.4. (2025). Available online at: https://readxl.tidyverse.org/ (Accessed Sep 4, 2025).

[24] Wickham H Averick M Bryan J Chang W Mcgowan L François R . Welcome to the tidyverse. JOSS. (2019) 4:1686. doi: 10.21105/joss.01686

[25] Firke S. *Janitor: Simple Tools for Examining and Cleaning Dirty Data*. R-Package Version 2.2.1.9000. (2024). Available online at: https://sfirke.github.io/janitor/.

[26] Tierney N Cook D. Expanding tidy data principles to facilitate missing data exploration, visualization and assessment of imputations. J Stat Softw. (2023) 105:1–31. doi: 10.18637/jss.v105.i0736798141

[27] Gohel D Moog S Heckmann M. *Officer: Manipulation of Microsoft Word and PowerPoint Documents. R-Package Version 0.6.10*. (2025). Available online at: https://ardata-fr.github.io/officeverse/.

[28] Kassambara A. *Ggpubr: “ggplot2” Based Publication Ready Plots. R-Package Version 0.6.1*. (2025). Available online at: https://rpkgs.datanovia.com/ggpubr/.

[29] Wickham H. ggplot2: Elegant graphics for data analysis. New York: Springer-Verlag (2016).

[30] Wilke CO. *Cowplot: Streamlined Plot Theme and Plot Annotations for ‘ggplot2’*. R package version 1.1.3. 2025; Available online at: https://wilkelab.org/cowplot/.

[31] Robin X Turck N Hainard A Tiberti N Lisacek F Sanchez J . pROC: an open-source package for R and S+ to analyze and compare ROC curves. BMC Bioinformatics. (2011) 12:77. doi: 10.1186/1471-2105-12-77, 21414208 PMC3068975

[32] Han MW Jeong SY Suh CH Park H Guenette JP Huang RY . Incidence of immune effector cell-associated neurotoxicity among patients treated with CAR T-cell therapy for hematologic malignancies: systematic review and meta-analysis. Front Neurol. (2024) 15:1392831. doi: 10.3389/fneur.2024.1392831 39474369 PMC11518750

[33] Neelapu SS Locke FL Bartlett NL Lekakis LJ Miklos DB Jacobson CA . Axicabtagene ciloleucel CAR T-cell therapy in refractory large B-cell lymphoma. N Engl J Med. (2017) 377:2531–44. doi: 10.1056/NEJMoa1707447, 29226797 PMC5882485

[34] Schuster SJ Bishop MR Tam CS Waller EK Borchmann P McGuirk JP . Tisagenlecleucel in adult relapsed or refractory diffuse large B-cell lymphoma. N Engl J Med. (2019) 380:45–56. doi: 10.1056/NEJMoa1804980, 30501490

[35] Muhsen IN Roloff GW Faramand R Othman T Valtis Y Kopmar NE . Outcomes of brexucabtagene autoleucel in patients with relapsed/refractory acute lymphoblastic leukemia with CNS involvement. Blood Adv. (2025) 9:4081–9. doi: 10.1182/bloodadvances.2024015779, 40334068 PMC12359224

[36] Munshi NC Anderson LD Shah N Madduri D Berdeja J Lonial S . Idecabtagene vicleucel in relapsed and refractory multiple myeloma. N Engl J Med. (2021) 384:705–16. doi: 10.1056/NEJMoa2024850 33626253

[37] Kamdar M Solomon SR Arnason J Johnston PB Glass B Bachanova V . Lisocabtagene maraleucel versus standard of care with salvage chemotherapy followed by autologous stem cell transplantation as second-line treatment in patients with relapsed or refractory large B-cell lymphoma (TRANSFORM): results from an interim analysis of an open-label, randomised, phase 3 trial. Lancet. (2022) 399:2294–308. doi: 10.1016/S0140-6736(22)00662-6, 35717989

[38] Usmani SZ Berdeja JG Jakubowiak A Agha M Cohen AD Madduri D . Updated results from the CARTITUDE-1 study of ciltacabtagene autoleucel, a B-cell maturation antigen–directed chimeric antigen receptor T cell therapy, in relapsed/refractory multiple myeloma. Hematol Transfus Cell Ther. (2021) 43:S272. doi: 10.1182/blood-2021-146060

[39] Rubin DB Al Jarrah A Li K LaRose S Monk AD Ali AB . Clinical predictors of neurotoxicity after chimeric antigen receptor T-cell therapy. JAMA Neurol. (2020) 77:1536–42. doi: 10.1001/jamaneurol.2020.2703, 32777012 PMC7418044

[40] Tan JY Yeo YH Kin HWK Ang QX Chisti MM Ezekwudo D . Sex differences in outcomes of chimeric antigen receptor (CAR) T-cell therapy. Cancer Med. (2025) 14:e70831. doi: 10.1002/cam4.70831, 40129265 PMC11933716

[41] Mauvais-Jarvis F Klein SL Levin ER. Estradiol, progesterone, immunomodulation, and COVID-19 outcomes. Endocrinology. (2020) 161:127. doi: 10.1210/endocr/bqaa127, 32730568 PMC7438701

[42] Lapidus AH Anderson MA Harrison SJ Dickinson M Kalincik T Lasocki A. Neuroimaging findings in immune effector cell associated neurotoxicity syndrome after chimeric antigen receptor T-cell therapy. Leuk Lymphoma. (2022) 63:2364–74. doi: 10.1080/10428194.2022.2074990, 35570737

[43] Schoeberl F Tiedt S Schmitt A Blumenberg V Karschnia P Burbano VG . Neurofilament light chain serum levels correlate with the severity of neurotoxicity after CAR T-cell treatment. Blood Adv. (2022) 6:3022–6. doi: 10.1182/bloodadvances.2021006144, 35042236 PMC9131908

