# Supplementary material for: The Berlin-Hannover ICANS severity assessment–a novel bedside test to evaluate CAR T-cell-associated neurotoxicity
Source: Front Neurol. 2026 Jan 21;17:1726779. doi: 10.3389/fneur.2026.1726779 (PMC12867840; doi:10.3389/fneur.2026.1726779)
Supplement: Supplementary file 1 [file Supplementary_file_1.docx]

Supplementary Material

# Supplementary Tables

## Supplementary Table 1

**Supplementary Table 1:** Baseline neurological findings from clinical examination and EEG. Data are presented as absolute and relative frequencies (%). EEG: Electroencephalography; ICANS: Immune Effector Cell-Associated Neurotoxicity Syndrome; ^#^Data on neurological examinations prior to the start of therapy were available for 80 patients; ^*^Mild hypesthesia of the left arm; ^**^Sensory-motor, right-sided incomplete paraplegia with bladder/rectal incontinence; ^***^ EEG data were available for 26 patients prior to the start of therapy.

| **Neurological findings at baseline** | **Total cohort (n = 100)^#^** | **Non-ICANS (n = 63)** | **ICANS (n = 37)** | **p-value** |
| --- | --- | --- | --- | --- |
| No pathological findings | 15 (15%) | 10 (15.9%) | 5 (13.5%) | 0.772 |
| Cranial nerve damage | 16 (16%) | 10 (15.9%) | 6 (16.2%) | 1.000 |
| Paresis | 10 (10%) | 6 (9.5%) | 4 (10.8%) | 1.000 |
| Tremor | 8 (8%) | 6 (9.5%) | 2 (5.4%) | 0.474 |
| Peripheral neuropathy | 56 (56%) | 30 (47.6%) | 26 (70.3%) | **0.045** |
| Other sensory deficits^*^ | 1 (1%) | 1 (1.6%) | 0 (0%) | 1.000 |
| Ataxia | 15 (15%) | 7 (11.1%) | 8 (21.6%) | 0.245 |
| Hyporeflexia/ Areflexia | 15 (15%) | 10 (15.9%) | 5 (13.5%) | 0.772 |
| Executive dysfunction/ cognitive dysfunction | 9 (9%) | 5 (7.9%) | 4 (10.8%) | 0.729 |
| Others^**^ | 1 (1%) | 1 (1.6%) | 0 (0%) | 1.000 |
| **EEG at baseline^***^** |  |  |  |  |
| No pathological findings | 18 (18%) | 13 (20.6%) | 5 (13.5%) | 0.640 |
| Increased cerebral excitability | 4 (4%) | 2 (3.2%) | 2 (5.4%) | 0.570 |
| Regional brain dysfunction (theta/delta slowing) | 3 (3%) | 2 (3.2%) | 1 (2.7%) | 1.000 |

## Supplementary Table 2

**Supplementary Table 2:** Tasks and point distribution of the ICE score. The ICE score ranges from 0 to 10. If there are no neurological abnormalities, the full score is awarded. One point is deducted from the total score for each subtest that is not passed. ICE: Immune Effector Cell-Associated Encephalopathy.

| **Domains** | **Tasks** | **Points** |
| --- | --- | --- |
| Orientation | Month | 1 |
|  | Year | 1 |
|  | City | 1 |
|  | Hospital | 1 |
| Object naming | Naming three objects | 3 |
| Handwriting | Writing a complete sentence | 1 |
| Attention | Continuous subtraction from 100 in increments of 10 (no points awarded for ≥ 1 error) | 1 |
| Ability to follow commands | Performing a gesture (e.g. show two fingers, close your eyes, stick out your tongue) | 1 |

## Supplementary Table 3

**Supplementary Table 3:** Tasks and point distribution of the BHISA score. The BHISA score ranges from 0 to 17. If there are no neurological abnormalities, the full score is awarded. One point is deducted from the total score for each subtest that is not passed. BHISA: Berlin-Hannover ICANS Severity Assessment. * Patient does not reach for the hands: 3 points, patient hesitates and asks what to do: 2 points, patient reaches for the hands the first time: 1 point, patient reaches for the hands the second time: 0 points

| **Domains** | **Tasks** | **Points** |
| --- | --- | --- |
| Orientation | Day | 1 |
|  | Month | 1 |
|  | Year | 1 |
|  | Place | 1 |
| Object naming | Two commonly used objects (e.g. ballpoint pen, glasses, gloves) are presented to the patient by the examiner with the request to name them. | 2 |
| Verbal fluency | Repeat a sentence: ‘According to yesterday's weather forecast, the sun is expected to shine this Sunday.’ | 1 |
| Handwriting | Rewrite the sentence: ‘The train departed on time.’ | 1 |
| Attention | Continuous substraction from 100 to 50 in increments of 10 (no points awarded for ≥ 1 error) | 1 |
| Executive/Frontal Function | Copy the cube | 1 |
|  | Applause sign | 1 |
|  | Environmental autonomy: The patient places their hands palm up. Without saying anything or looking at the patient, the examiner touches their palms. If the patient spontaneously reaches for their hands: ‘Please do not grab my hands’ (2x repetitions) | 3* |
|  | Luria sequence | 1 |
| Motor skills | Pronator drift test for 10 seconds (no points, if patient presents tremor) | 1 |
|  | Palmomental reflex | 1 |

# Supplementary Figures

## Supplementary Figure 1a-d


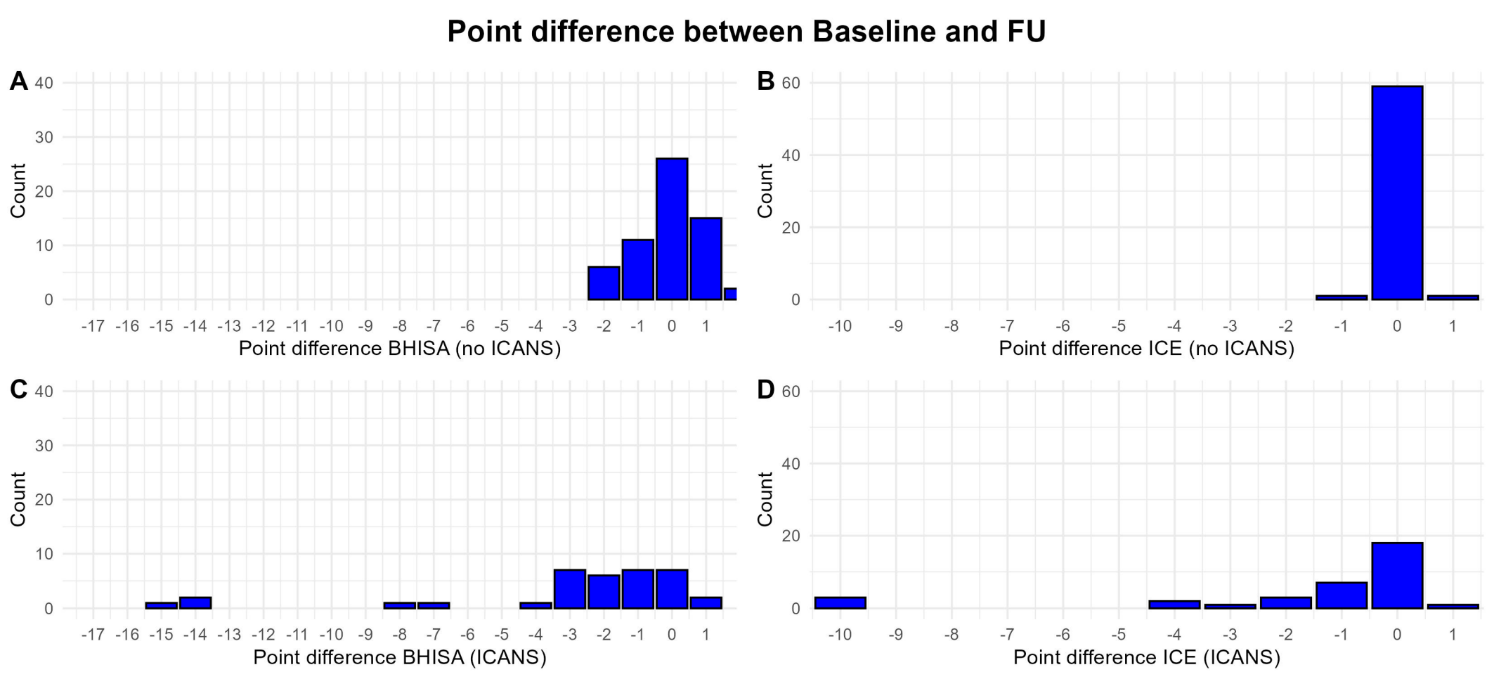


**Supplementary Figure 1A-D:** Distribution of ICE and BHISA score changes from baseline to FU (Δ = FU−baseline) in patients with ICANS and without ICANS. Negative values indicate a decrease from baseline. ICANS: Immune Effector Cell-Associated Neurotoxicity Syndrome; ICE: Immune Effector Cell-Associated Encephalopathy Score; BHISA: Berlin-Hannover ICANS Severity Assessment; FU: Follow-up

## Supplementary Figure 2 a-d


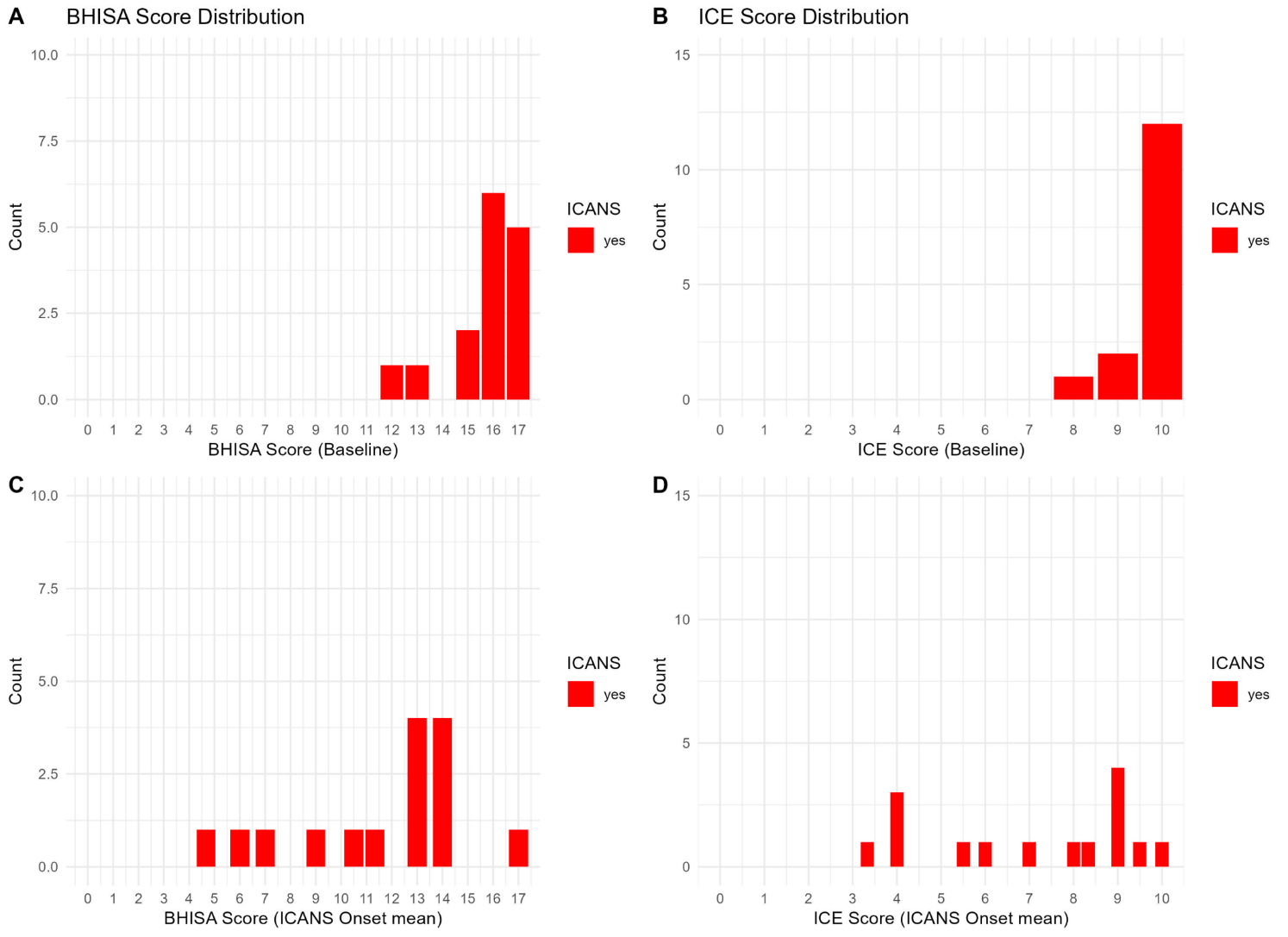


**Supplementary Figure 2 A-D:** Distribution of BHISA scores (A, C) and ICE scores (B, D) at baseline (prior to CAR-T infusion) and at the onset of ICANS. When multiple assessments were available during an ICANS episode, mean scores are shown. BHISA and ICE data at ICANS onset were available for 15 patients. ICE: Immune Effector Cell-Associated Encephalopathy Score; BHISA: Berlin-Hannover ICANS Severity Assessment; ICANS: Immune Effector Cell-Associated Neurotoxicity Syndrome

## Supplementary Figure 3 a-d


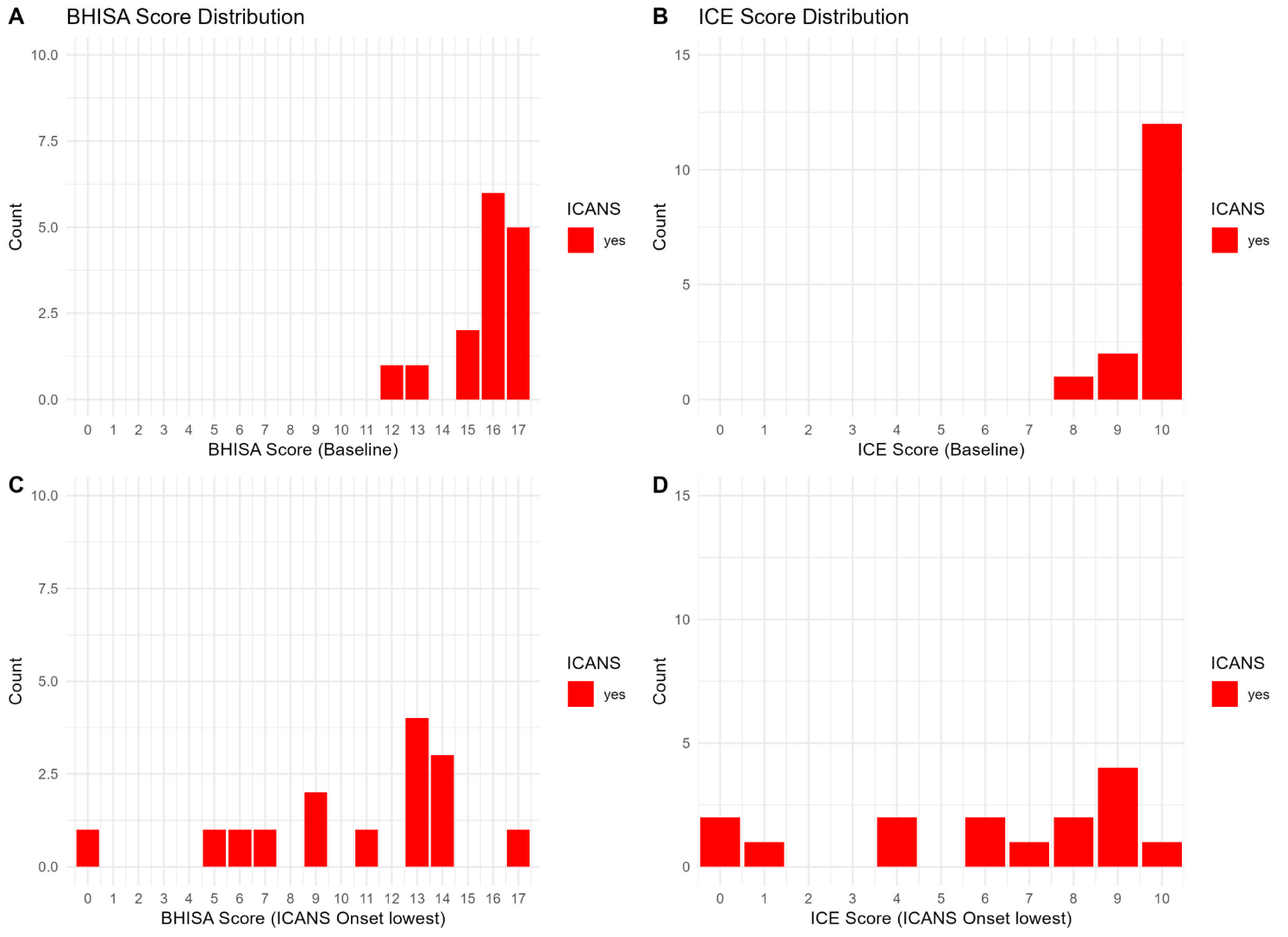


**Supplementary Figure 3 A-D:** Distribution of BHISA scores (A, C) and ICE scores (B, D) at baseline (prior to CAR-T infusion) and at ICANS onset. When multiple assessments were performed during an ICANS episode, the lowest score was used. BHISA and ICE data at ICANS onset were available for 15 patients. ICE: Immune Effector Cell-Associated Encephalopathy Score; BHISA: Berlin-Hannover ICANS Severity Assessment; ICANS: Immune Effector Cell-Associated Neurotoxicity Syndrome

## Supplementary Figure 4


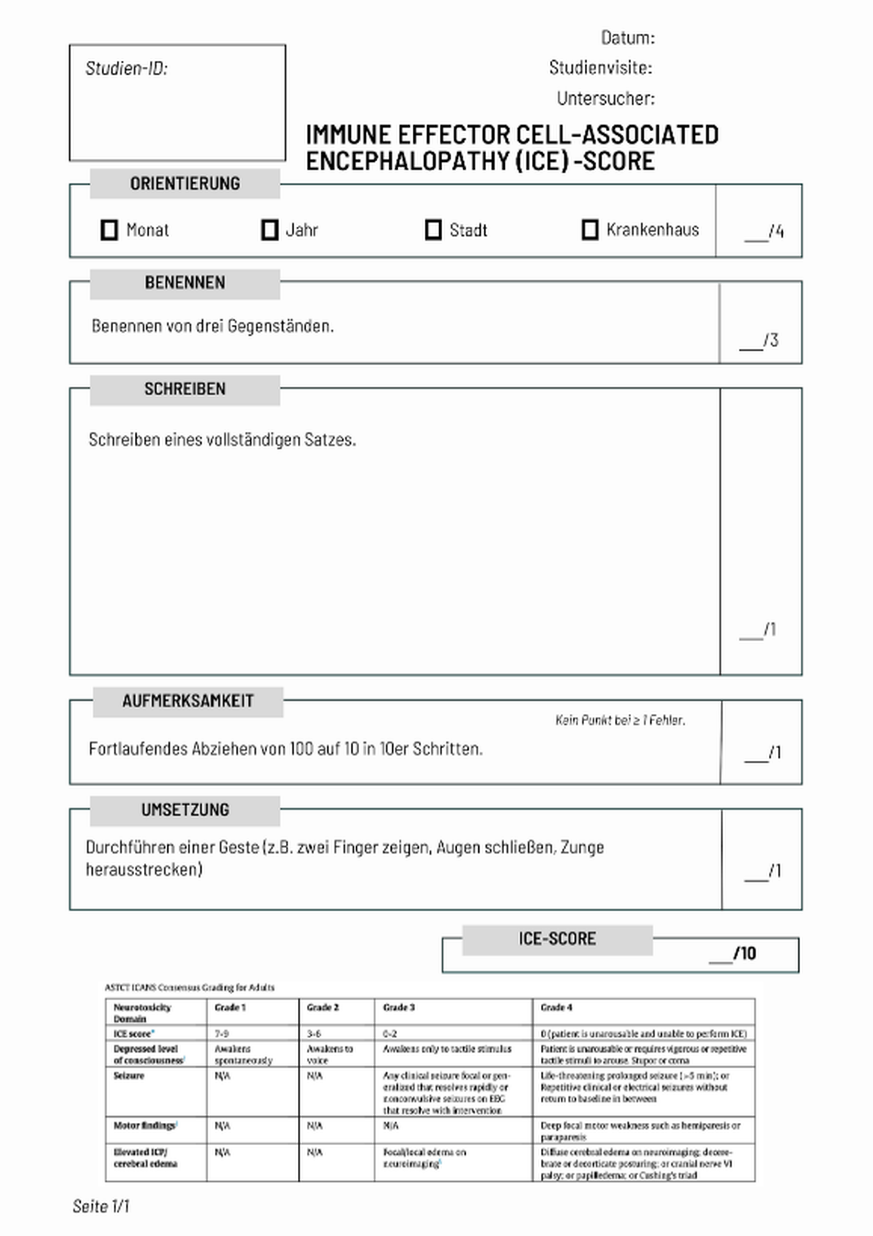


**Supplementary Figure 4:** German task sheets for the ICE score. The ICE score ranges from 0 to 10. If there are no neurological abnormalities, the full score is awarded. One point is deducted from the total score for each subtest that is not passed. In the orientation section, one point is awarded for each correct answer and one point for each correctly named object. ICE: Immune Effector Cell-Associated Encephalopathy.

## Supplementary Figure 5


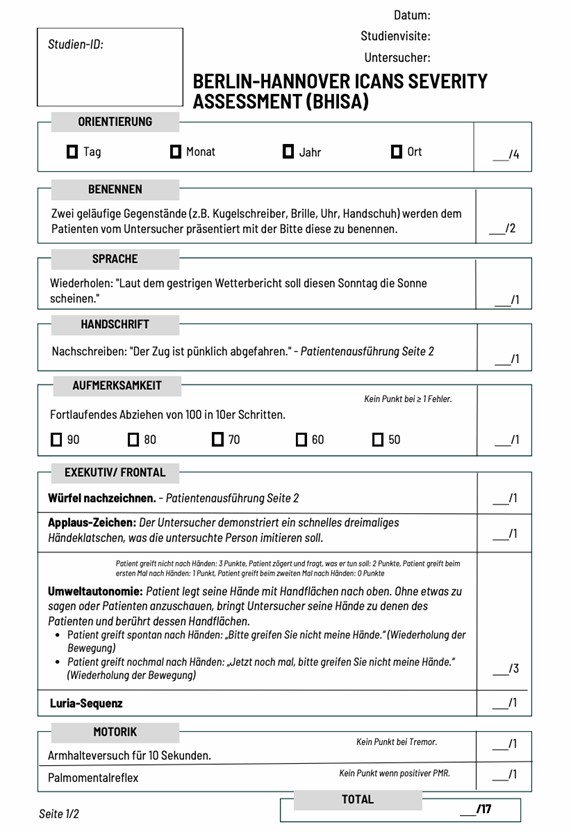


**Supplementary Figure 5**: German task sheets for the BHISA score. The BHISA score ranges from 0 to 17. If there are no neurological abnormalities, the full score is awarded. One point is deducted from the total score for each subtest that is not passed. In the orientation section, one point is awarded for each correct answer and one point for each correctly named object. BHISA: Berlin-Hannover ICANS Severity Assessment
